# Rapid Genomic and Genetic Changes in the First Generation of Autotetraploid Lineages Derived from Distant Hybridization of *Carassius auratus* Red Var. (♀) × *Megalobrama amblycephala* (♂)

**DOI:** 10.1007/s10126-018-9859-8

**Published:** 2018-11-13

**Authors:** Qinbo Qin, Liu Cao, Yude Wang, Li Ren, Qiwen Liu, Yuwei Zhou, Chongqing Wang, Huan Qin, Chun Zhao, Shaojun Liu

**Affiliations:** State Key Laboratory of Developmental Biology of Freshwater Fish, College of Life Sciences, Hunan Normal University, Changsha, 410081 Hunan People’s Republic of China

**Keywords:** Autotetraploid lineages, Chromosomal locus, Genome, Transcriptome, Meiosis

## Abstract

Autopolyploids are traditionally used to demonstrate multivalent pairing and unstable inheritance. However, the autotetraploid fish (4nRR) (RRRR, 4n = 200) derived from the distant hybridization of *Carassius auratus* red var. (RCC) (RR, 2n = 100) (♀) × *Megalobrama amblycephala* (BSB) (BB, 2n = 48) (♂) exhibits chromosome number (or ploidy) stability over consecutive generations (*F*_1_–*F*_10_). Comparative analysis based on somatic and gametic chromosomal loci [centromeric, 5S rDNA, and Ag-NORs (silver-stained nucleolar organizer regions)] revealed that a substantial loss of chromosomal loci during genome doubling increases the divergence between homologous chromosomes and that diploid-like chromosome pairing was restored during meiosis in the first generation of 4nRR lineages. In addition, a comparative analysis of genomes and transcriptomes from 4nRR (*F*_1_) and its diploid progenitor (RCC) exhibited significant genomic structure and gene expression changes. From these data, we suggest that genomes and genes diverge and that expression patterns change in the first generations following autotetraploidization, which are processes that might contribute to the stable inheritance and successful establishment of autotetraploid lineages.

**Electronic supplementary material:**

The online version of this article (10.1007/s10126-018-9859-8) contains supplementary material, which is available to authorized users.

## Introduction

Polyploidy has played an important role in the evolutionary history of vertebrates and other eukaryotes (Masterson 1994; Comai 2005; Mallet 2007; Otto 2007; Rieseberg and Willis 2007; Wood et al. 2009). Because an additional set (or sets) of chromosomes may originate from the same or different species, polyploids have been classified into two major categories: autopolyploids and allopolyploids (Comai 2005; Otto 2007). The former shows multivalent pairing during meiosis, whereas the latter predominantly exhibits bivalent pairing (Jackson 1982; Ramsey and Schemske 2002; Parisod 2010). Multivalent pairing can cause meiotic irregularities and result in reduced fertility compared with the diploid progenitors (Parisod 2010). Until recently, autopolyploids were believed to suffer from several evolutionary disadvantages compared with allopolyploids and were considered rare evolutionary dead-ends (Otto 2007; Soltis and Soltis 2010). However, accumulating evidence indicates that the actual appearance of autotetraploid species might be significantly underestimated and that autopolyploidy might contribute more to evolution and species diversification than traditionally thought (Barker et al. 2016).

Hybridization is one of the primary mechanisms for the origin of species leading to the formation of allopolyploids (Otto and Whitton 2000; Soltis and Soltis 2000; Liu 2010). The pairing of homologous chromosomes is defective in *F*_1_ hybrids (diploids) because of divergence in the number and structure of chromosomes, but each homologous chromosome can have its own pairing partner through genome doubling in somatic or germ cells (Wu et al. 2001; Paterson et al. 2004). In our previous research, fertile allotetraploid hybrids (4nRB) (RRBB, 4n = 148) were obtained in the first generation of RCC (RR, 2n = 100) (**♀**) × BSB (BB, 2n = 48) (**♂**); these hybrids exhibited abnormal chromosomal behavior during meiosis and generated autodiploid gametes with two sets of RCC-derived chromosomes (RR, 2n = 100) (Liu et al. 2007; Qin et al. 2014a, 2015a). Thus, autodiploid sperm can fertilize autodiploid ova, resulting in 4nRR formation (RRRR, 4n = 200) (Qin et al. 2014b). During consecutive generations (*F*_1_–*F*_10_), the 4nRR individuals exhibited normal chromosomal behavior during meiosis and produced diploid gametes, ensuring chromosome number stability. The consequences of chromosome doubling have been widely studied in allopolyploids (Pontes et al. 2004; Skalická et al. 2005; Xiong et al. 2011), whereas more limited data are available for autopolyploids. Thus, the autotetraploid lineages provide a model system to study genetic and genomic changes contributing to diploidization processes in autopolyploids.

In this study, we characterized the genomic and gene expression changes in early generations of autotetraploid lineages by comparing 4nRR (F_1_) and their diploid progenitors (RCC) using chromosomal locus, genome, and transcriptome analyses. Our study reveals significant genomic and gene expression alterations in the first generation after autotetraploid formation.

## Material and Methods

### Ethics

All the fish were cultured in ponds at the Protection Station of Polyploid Fish, Hunan Normal University, and fed with artificial feed. Fish treatments were performed according to the Care and Use of Agricultural Animals in Agricultural Research and Teaching, approved by the Science and Technology Bureau of China. Approval from the Department of Wildlife Administration was not required for the experiments conducted in this study. Fish were deeply anesthetized with 100 mg/L MS-222 (Sigma-Aldrich) before dissection.

### Animals and Crossing Procedure

During the reproductive seasons (April to June) of 2004–2006, 4nRB were produced by crossing RCC (♀) × BSB (♂). During the 2007 reproductive season (April to June), 4nRR were produced by 4nRB selfing. 2nG and 2nH were obtained through the artificial gynogenesis of 4nRB and 4nRR eggs, respectively. 4nRB and 4nRR eggs were activated with ultraviolet (UV)-treated, sterilized BSB sperm without treatment for chromosome doubling. During the reproductive seasons (April to June) of 2008–2016, *F*_2_–*F*_10_ autotetraploid fish were created in succession.

### Preparation of Chromosome Spreads

To obtain mitotic chromosome spreads, preparations were made from kidney tissues from 10 individuals each of RCC, BSB, 4nRB, 4nRR, 2nG, and 2nH at 1 year of age. For each type of fish, 200 metaphase chromosome spreads (20 spreads from each sample) were counted and analyzed. The preparations were examined under an oil-immersion lens at a magnification of × 3330.

### Fluorescence In Situ Hybridization

Two probes were used for fluorescence in situ hybridization (FISH): a species-specific centromere probe that was designed using the RCC genome and amplified by PCR using the primers 5′-TTCGAAAAGAGAGAATAATCTA-3′ and 5′-AACTCGTCTAAACCC GAACTA-3′, and a 5S gene probe that was constructed using the RCC genome and amplified by PCR using the primers 5′-GCTATGCCCGATCTCGTCTGA-3′ and 5′-CAGGTTGGTATGGCCGTAAGC-3′ (Qin et al. 2010). The species-specific centromere probes were labeled with biotin-16-dUTP (using a biotin-nick translation kit, Roche, Germany), and the 5S gene probes were labeled with DIG-11-dUTP (using a DIG-nick translation kit, Roche, Germany). FISH was performed according to He et al. (2012). Two hundred metaphase chromosome spreads from ten individuals were analyzed for each type of fish (RCC, BSB, 4nRB, 4nRR, 2nG, and 2nH). Preparations were examined under an inverted microscope (CW4000, Leica, Germany) with a confocal imaging system (LCS SP2, Leica). Captured images were colored and superimposed in Adobe Photoshop CS6.

### Silver Nitrate Staining

Silver nitrate staining (Ag-NOR staining) was performed according to the following steps. First, air-dried chromosomes were fixed on slides. Then, slides were incubated in silver nitrate solution (2 g gelatin in 100 mL 1% formic acid with two parts 50% silver nitrate solution in distilled water) for 3 min at 60 °C in the dark. The slides were then washed in deionized water. Two hundred metaphase chromosome spreads (20 spreads in each sample) were randomly counted and analyzed under an oil immersion lens.

### Identification of SNPs and Variations Using DNA Re-sequencing

DNA libraries were constructed with an insert size of ~ 500 bp according to the manufacturer’s instructions (Illumina, San Diego, CA, USA). Libraries were sequenced using the Illumina HiSeq 2500 platform (paired-end 125 bp reads). A total of 819,330,524 reads (103 Gb) were generated from three 4nRR individuals (Table S1). Approximately 34 Gb of clean reads were recovered for each sample, yielding a minimum of 10-fold genomic coverage. Then, we removed the pair-end reads containing ≥ 5% unidentified nucleotides (N), the adapter sequences, and low-quality reads. Sequences were submitted to GenBank with the accession numbers SRX2981769, SRX2981275, and SRX2992432. The high-quality reads were mapped to the RCC reference genome using BWA software with default options (Li and Durbin 2010). The reference genome of RCC was downloaded from DDBJ/EMBL/GenBank (Accession NO. LGRG00000000). We removed PCR duplicates and resynchronized mate information using Mark-Duplicates and Fix Mate Information in the Picard software package (version 1.48, http://broadinstitute.github.io/picard/), respectively.

Local alignment around indels was performed on de-duplicated reads using Realigner Target Creator and Indel Realigner in the Genome Analysis Toolkit (GATK; version 3.5) (Altshuler et al. 2010). Multi-sample SNP genotyping was performed to identify SNPs using the Unified Genotyper in GATK. To reduce the false discovery rate (FDR), we performed hard filtering based on the following criteria: QUAL < 30.0, QD < 2.0, MQ < 40.0, FS > 60.0, HaplotypeScore > 13.0, MQRankSum < − 12.5, and ReadPosRankSum < − 8.0. All the filtered SNPs were annotated to 15 functional categories using snpEff (http://snpeff.sourceforge.net/). Then, the software BreakDancer (version 1.1) was used to detect SVs in the autotetraploid genome. There were three types of SVs detected in the genome, including INS, DEL, and INV (Table S3). To obtain the function distribution of SNPs and SVs in coding sequences, functional enrichment analysis was performed based on the genome annotation results (Ye et al. 2006).

### Transcriptome Reconstruction, Annotation, and Analysis

4nRR and RCC ovary at the same developmental stage were stored in RNAlater (Ambion, Carlsbad, CA, USA) at − 80 °C. After removing RNAlater, total RNA was purified using Trizol Reagent (Ambion, Carlsbad CA92008, USA) and quantified with an Agilent 2100 Bioanalyzer (Agilent, Santa Clara, CA, USA). 4nRR and RCC sequencing libraries were then constructed and sequenced using an Illumina HiSeq™ 4000 (Illumina, San Diego, CA, USA). Adapters and low-quality reads were removed using a strict screening procedure. De novo transcriptome assembly was performed using Trinity (Dion-Côté et al. 2014). Several databases, including NR, Swiss-Prot, KEGG, and COG, were used for annotation.

To calculate relative expression levels, we used the FPKM method (RSEM) and Li and Dewey (2011). The threshold *P* value was determined using the FDR in multiple tests and analyses. Genes were considered differentially expressed when *q* value < 0.005 and |log2 (foldchange)| > 1. Blast2GO v2.5.0 (Conesa et al. 2005) was used for GO enrichment analyses, which provided functional annotation of differentially expressed genes. GO terms were considered significantly enriched at FDR ≤ 0.05 (SRR5874930, SRR5877247).

### Data Availability Statement

All sequence and the complete clean reads for those libraries have been uploaded to the NCBI Sequence Read Archive site (http://www.ncbi.nlm.nih.gov/sra/; accession nos. GO485556, JQ086761, SRX2981769, SRX2981275, SRX2992432, SRR5874930, and SRR5877247).

## Results

### Creation of Experimental Fish

**Fig. 1:**
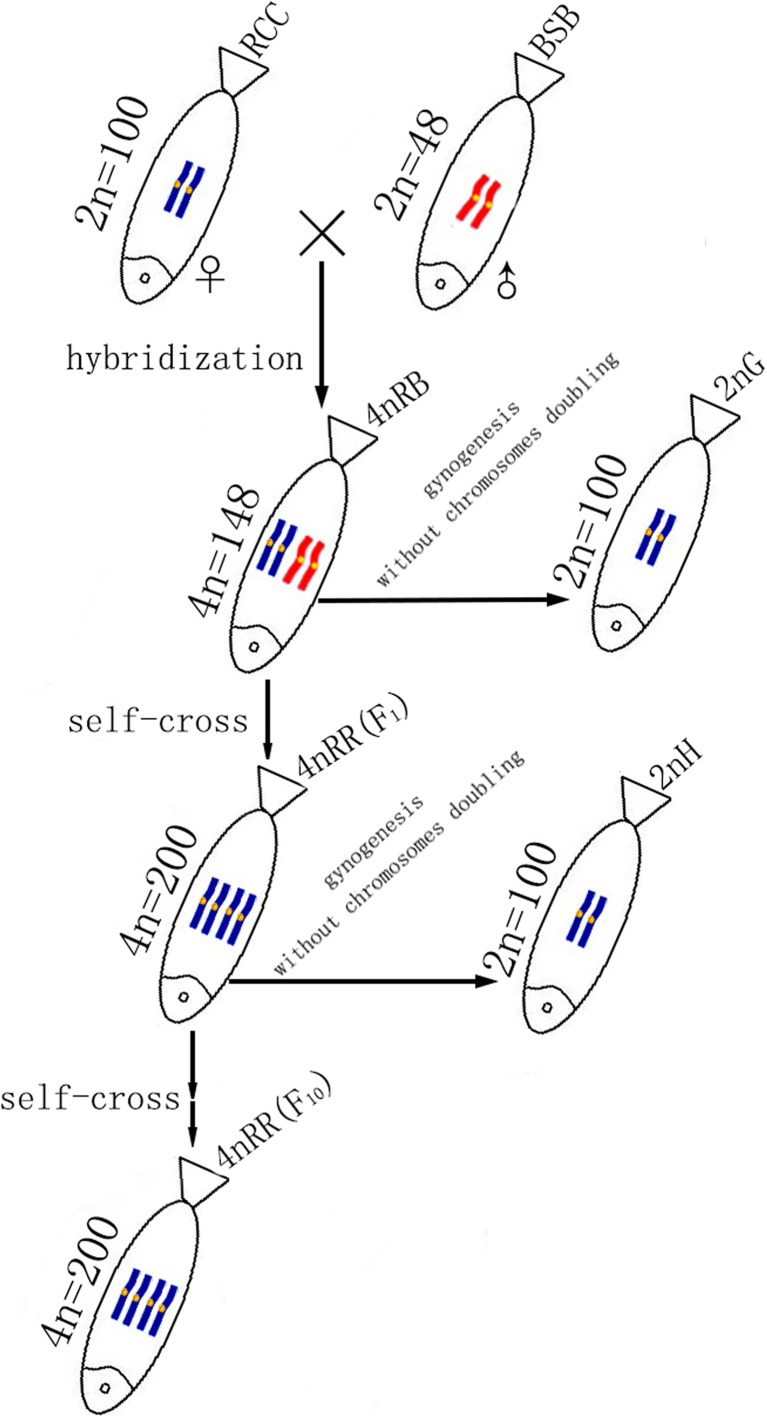
Formation of experimental fish hybrids. RCC, *Carassius auratus* red var.; BSB, *Megalobrama amblycephala*; 4nRB, allotetraploid hybrid; 2nG, gynogenetic progenies of allotetraploid hybrid; 4nRR, autotetraploid hybrid; 2nH, gynogenetic progenies of autotetraploid hybrid

4nRB (RRBB, 4n = 148) fish were obtained in the first generation of the cross RCC (RR, 2n = 100) (♀) × BSB (BB, 2n = 48) (♂), which can produce autodiploid gametes (2n = 100, RR). Subsequently, 4nRR (RRRR, 4n = 200) individuals were produced by self-crossing 4nRB, and 4nRR lineages (*F*_1_–*F*_10_) were successively formed (Fig. 1). To understand the genomic changes that occur during genome doubling, autodiploid gynogenetic progeny (2nG) (RR, 2n = 100) was produced by artificial gynogenesis from autodiploid 4nRB eggs (RR, 2n = 100) that were activated with UV-treated sterilized BSB sperm without treatment for chromosome doubling (Fig. 1). To precisely determine the occurrence of diploid-like chromosome pairing during meiosis in 4nRR (F_1_), gynogenetic progeny (2nH) (RR, 2n = 100) was produced by artificial gynogenesis from diploid eggs (2n = 100) of 4nRR that were activated with UV-treated sterilized BSB sperm without treatment for chromosome doubling (Fig. 1).

### Genomic Changes in Newly Established Autotetraploid Genomes

**Fig. 2:**
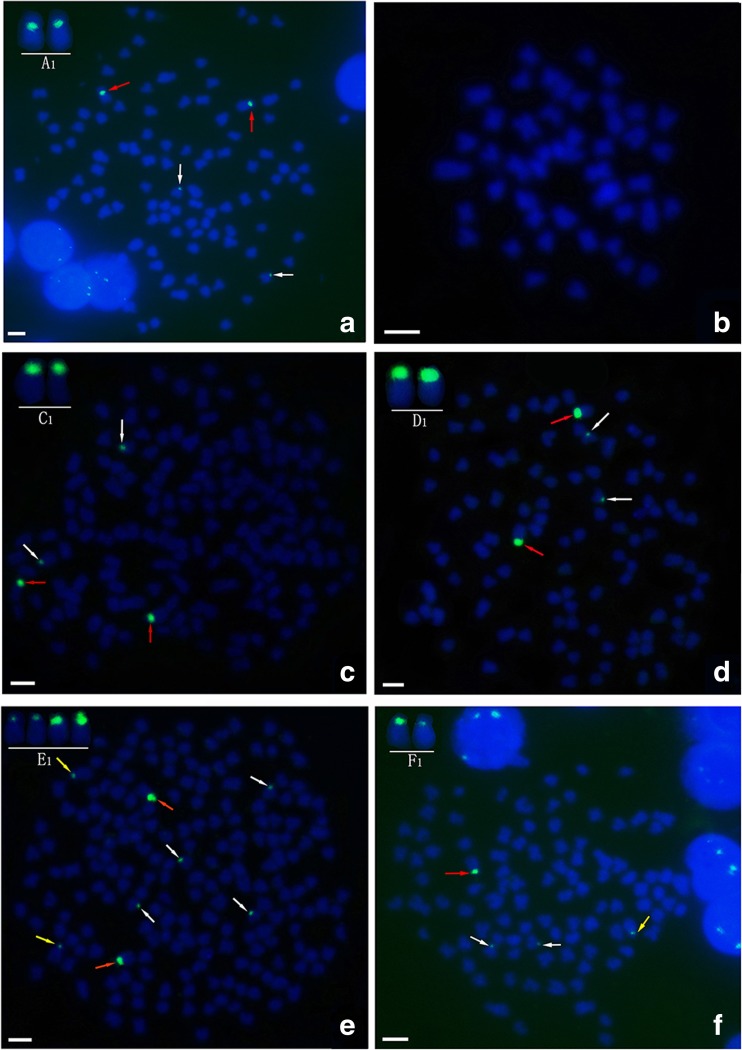
FISH hybridization signals in the metaphase chromosomes of RCC, BSB, 4nRB, 2nG, 4nRR and 2nH with 5S rDNA probe. **a** The two large 5S rDNA gene loci (red arrows) and the two small 5S rDNA gene loci (white arrows) in RCC are shown. A1 in **a**, C1 in **c**, and D1 in **d** indicate that the two large 5S rDNA gene loci were located on a pair of homologous submetacentric chromosomes. **b** No 5S gene locus is found in BSB. **c** The two large 5S rDNA gene loci (red arrows) and the two small 5S rDNA gene loci (white arrows) in 4nRB are shown. **d** The two large 5S rDNA gene loci (red arrows) and two small 5S rDNA gene loci (white arrows) in 2nG are shown. **e** The two large 5S rDNA gene loci (red arrows) and the six small 5S rDNA gene loci (white and yellow arrows) in 4nRR are shown, E1 indicates that two large 5S rDNA gene loci (red) and two small 5S rDNA gene loci (yellow) located on two pairs of homologous submetacentric chromosome, respectively. **f** A large 5S rDNA gene loci (red arrows) and the three small 5S rDNA gene loci (white and yellow arrows) in 2nH are shown. F1 indicates that a large 5S rDNA gene loci (red) and a small 5S rDNA gene loci (yellow) were located on a pair of homologous submetacentric chromosomes. Bars in **a**–**f**: 3 μm. RCC, *Carassius auratus* red var.; BSB, *Megalobrama amblycephala*; 4nRB, allotetraploid hybrid; 2nG, gynogenetic progenies of allotetraploid hybrid; 4nRR, autotetraploid hybrid; 2nH, gynogenetic progenies of autotetraploid hybrid

Using *5S rDNA* (340 bp; sequence number: GQ485556) as a probe, a pair of large *5S rDNA* loci was identified on homologous submetacentric chromosomes, while a pair of small *5S rDNA* loci was localized to homologous subtelocentric chromosomes (Fig. 2a and Table 1). The *5S rDNA* locus was not detected in BSB (Fig. 2b and Table 1). The *5S rDNA* loci of 4nRB and 2nG were both similar to RCC (Fig. 2c, d, Table 1), suggesting that the autodiploid gamete of 4nRB has a pair of large *5S rDNA* loci on a homologous submetacentric chromosome and a pair of small *5S rDNA* loci on a homologous subtelocentric chromosome. Thus, two pairs of large *5S rDNA* loci on homologous submetacentric chromosomes were expected in 4nRR (*F*_1_). Although two pairs of *5S rDNA* loci on homologous submetacentric chromosomes were found in 4nRR (F_1_), one pair of large *5S rDNA* loci on the submetacentric chromosome was transformed to small loci (Fig. 2e, yellow arrow and Table 1). In the 4nRR individuals (*F*_7_–*F*_10_), only one pair of large *5S rDNA* loci on the submetacentric chromosome was observed; the other pair of *5S rDNA* loci on the submetacentric chromosome had completely disappeared (Table 1).

**Table 1:** Examination of chromosomal locus number

Fish type^a^	No. of fish	No. of metaphase	Ag-NORs	Centromeric	5s rDNA	
			No. of loci	No. of loci	No. of big loci	No. of small loci
RCC	10	200	4	100	2	2
BSB	10	200	No test	0	0	0
4nRB	10	200	No test	100	2	2
2nG	10	200	4	100	2	2
4nRR (*F*_1_)	10	200	4	100	2	6
2nH	10	200	2	50	1	3
4nRR (*F*_7_)	10	200	4	100	2	4

^a^RCC, *Carassius auratus* red var.; BSB, *Megalobrama amblycephala*; 4nRB, allotetraploid hybrid; 2nG, gynogenetic progenies of allotetraploid hybrid; 4nRR, autotetraploid; 2nH, gynogenetic progenies of autotetraploid

**Fig. 3:**
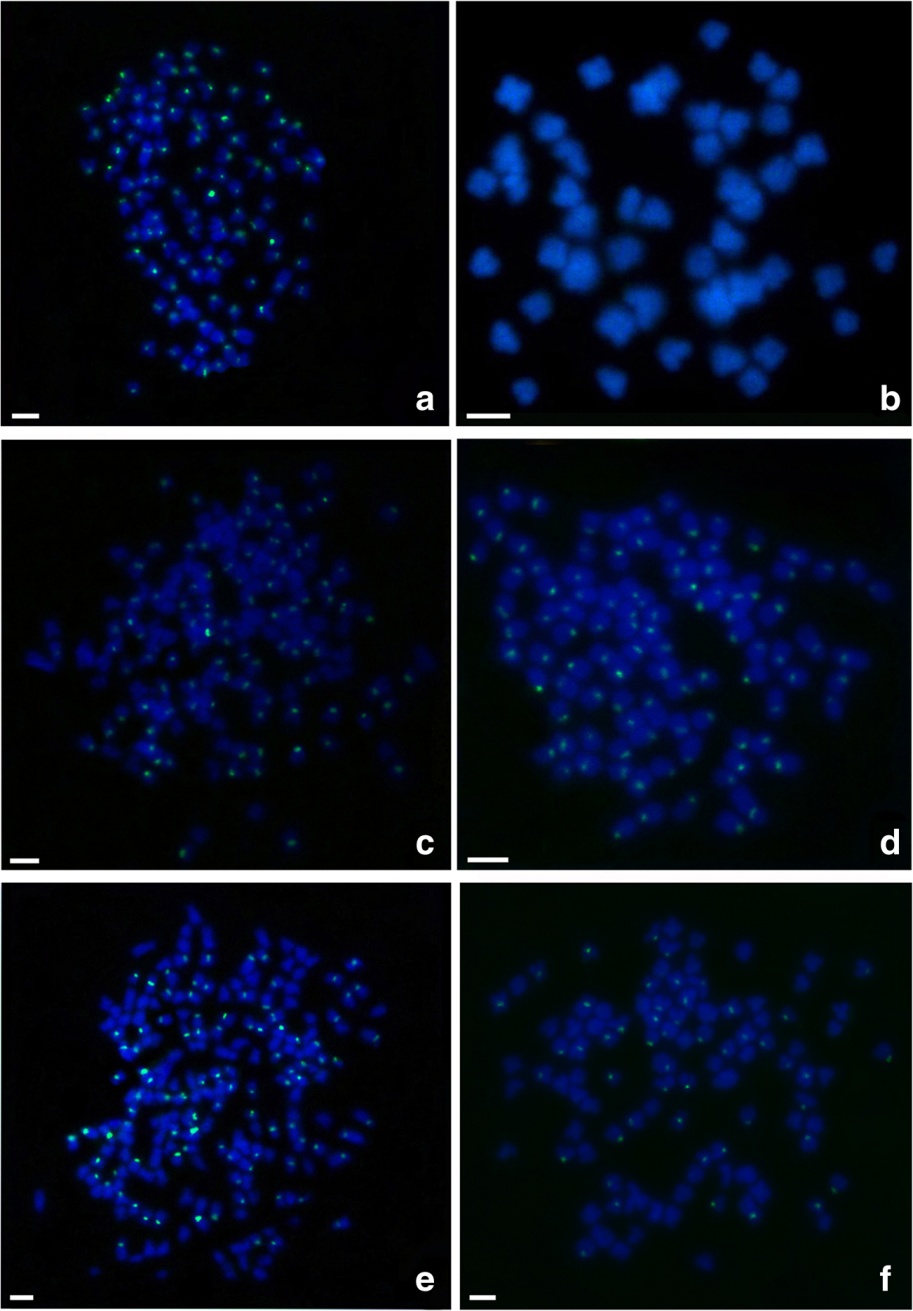
FISH hybridization signals in the metaphase chromosomes of RCC, BSB, 4nRB, 2nG, 4nRR, and 2nH with species-specific centromere probe. **a** The centromere probe hybridized to 100 chromosomes in RCC. **b** No chromosome hybridized is found in BSB. **c**–**e** The centromere probe hybridized to 100 RCC-derived chromosomes in 4nRB, 2nG, 4nRR, respectively. **f** The centromere probe hybridized to 50 chromosomes of 2nH. Bars in **a**–**f**: 3 μm. RCC, *Carassius auratus* red var.; BSB, *Megalobrama amblycephala*; 4nRB, allotetraploid hybrid; 2nG, gynogenetic progenies of allotetraploid hybrid; 4nRR, autotetraploid hybrid; 2nH, gynogenetic progenies of autotetraploid hybrid

**Fig. 4:**
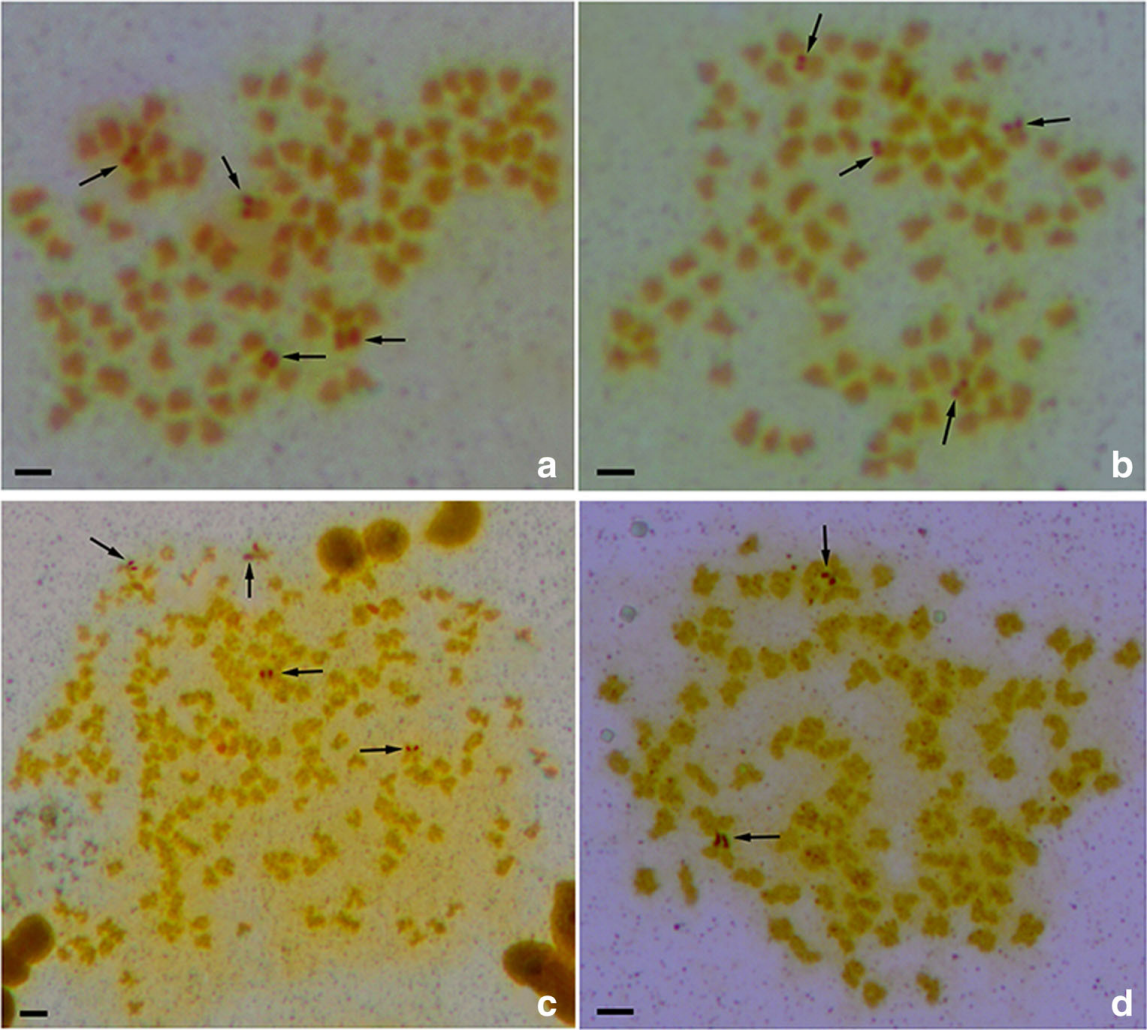
Ag-NORs in the metaphase chromosomes of RCC, 2nG, 4nRR, and 2nH. **a**–**c** The four Ag-NORs in RCC, 2nG, 4nRR. **d** The two Ag-NORs in 2nH. Bars in **a**–**d** 3 μm. RCC, *Carassius auratus* red var.; 2nG, gynogenetic progenies of allotetraploid hybrid; 4nRR, autotetraploid hybrid; 2nH, gynogenetic progenies of autotetraploid hybrid

The species-specific centromere probe (repetitive sequences of 263 bp; sequence number: JQ086761) hybridized to 100 chromosomes in RCC individuals (Fig. 3a and Table 1) but did not hybridize to any chromosomes in BSB (Fig. 3b and Table 1). As expected, 100 chromosomal loci were found in 4nRB and 2nG (Fig. 3c, d, Table 1), suggesting that autodiploid 4nRB gametes have 100 chromosomal loci. Unexpectedly, only 100 chromosomal loci were observed in 4nRR individuals (*F*_1_) rather than the expected number of 200 (Fig. 3e and Table 1). In addition, four Ag-NORs were identified in RCC (Fig. 4a and Table 1) and 2nG (Fig. 4b and Table 1) individuals. Eight Ag-NORs were expected in 4nRR (*F*_1_), but only four Ag-NORs were found (Fig. 4c and Table 1).

Substantial losses of chromosomal loci (centromeric, *5S rDNA*, and Ag-NORs) were observed in the first generations of the autotetraploid lineages, indicating that rapid and widespread genomic changes occur in newly established autotetraploid genomes.

### Diploid-Like Chromosome Pairing in Autotetraploid Lineages

Each generation of 4nRR lineages possessed 200 chromosomes and produced diploid gametes with 100 chromosomes (Table 2), suggesting chromosome number stability during consecutive generations.

**Table 2:** Examination of chromosome number in autotetraploid lineages (*F*_1_–*F*_10_)

Fish type	No. of metaphase	Distribution of chromosome number (somatic cell)		No. of metaphase	Distribution of chromosome number (gamete)	
		< 200	200		< 100	100
*F*_1_	200	23	177	200	14	186
*F*_2_	200	28	172	200	10	190
*F*_3_	200	31	169	200	12	188
*F*_4_	200	24	176	200	18	182
*F*_5_	200	35	165	200	25	175
*F*_6_	200	33	167	200	21	179
*F*_7_	200	29	171	200	13	187
*F*_8_	200	38	162	200	11	189
*F*_9_	200	20	180	200	18	182
*F*_10_	200	36	164	200	22	178

Using *5S rDNA* (340 bp) as a probe, one large *5S rDNA* locus and one small *5S rDNA* locus were identified on the submetacentric chromosome in 2nH (Fig. 2f and Table 1), and 4nRR had a pair of large and a pair of small *5S rDNA* loci on the submetacentric chromosome (Fig. 2e). In addition, 4nRR (*F*_1_) contained 100 centromere loci (Fig. 3e) and four Ag-NORs (Fig. 4c), but only 50 centromere loci (Fig. 3f and Table 1) and two Ag-NORs (Fig. 4d and Table 1) were observed in 2nH. Comparative analysis of these somatic and gamete chromosomal loci in 4nRR (F_1_) revealed obvious chromosomal locus number halving during meiosis, indicating that diploid-like chromosome pairing was restored in the first generations of the autotetraploid lineages.

### Identification of SNPs and Structural Variation in Newly Established Autotetraploid Genomes

After re-sequencing 4nRR (F_1_), we obtained 819.33 million (M) clean reads (103.22 Gb) (Table S1). Then, we performed single-nucleotide polymorphism (SNP) calling and identified 15.90 M SNPs between 4nRR and the RCC reference genome (Table 3). SNPs were classified as either transitions (8.52 M, 53.64%) or transversions (7.37 M, 46.36%) (Table 3). We identified 1.31 M (8.26%) SNPs in coding sequences, of which 428,034 (2.69%) and 882,709 (5.55%) coding SNPs were categorized as non-synonymous and synonymous nucleotide substitutions, respectively (Table 3). The top 1000 genes containing the highest number of non-synonymous SNPs (nsSNPs) were related to anatomical structure development (88 genes, GO: 0048856), multicellular organismal development (85 genes, GO: 0007275), regulation of biological process (71 genes, GO: 0050789), and regulation of cellular process (66 genes, GO: 0050794), among others (Table S2). Comparing 4nRR and RCC, we calculated heterozygosity and homozygosity based on SNP type. More SNPs were heterozygous (12.22 M, 76.82%) with respect to the RCC genome, although 3.69 M SNPs were identified as homozygous (Table 3).

**Table 3:** The summary of SNPs in 4nRR (*F*_1_)

	Mapping to RCC	Ratio
SNP number	15,904,360	100.00%
Transition	8,531,142	53.64%
Transversion	7,373,218	46.36%
Heterozygosity	12,218,490	76.82%
Homozygosity	3,685,870	23.18%
Synonymous coding	882,709	5.55%
Non-synonymous coding	428,034	2.69%
Start lost	486	0.003%
Start gained	7542	0.05%
Stop gained	1481	0.01%
Stop lost	405	0.003%
Non-coding exon	2,436,084	15.32%
UTR 5′	41,746	0.26%
UTR 3′	218,005	1.37%
Intron	6,383,288	40.14%
Intergenic	5,205,128	32.73%
Splice site acceptor	709	0.004%
Splice site donor	975	0.01%
Splice site region	96,901	0.61%
Other	200,867	1.26%

*UTR* untranslated region

Structural variation (SV) analysis of 4nRR indicated that three types of SV were detected, with 89,575 insertions (INS), 42,472 deletions (DEL), and 4765 inversions (INV) identified by mapping to the RCC genome (Table S3). We performed an analysis of INS and DEL (merge as InDels) and obtained 5.66 M InDels with lengths ranging from 1 to 30 bp (Table S4). Among these InDels, only 44,271 (0.78%) were located in coding sequences, and 35.44% of these were in multiples of 3 bp. We performed GO enrichment of the top 1000 genes containing the greatest numbers of InDels (> 423 InDels in a gene) and found that they were mainly enriched in terms (molecular function) related to sequence-specific DNA binding (GO: 0043565), metal ion binding (GO: 0046872), and ATP binding (GO: 0005524).

### Significant Transcriptome Alteration in Autotetraploids

**Fig. 5:**
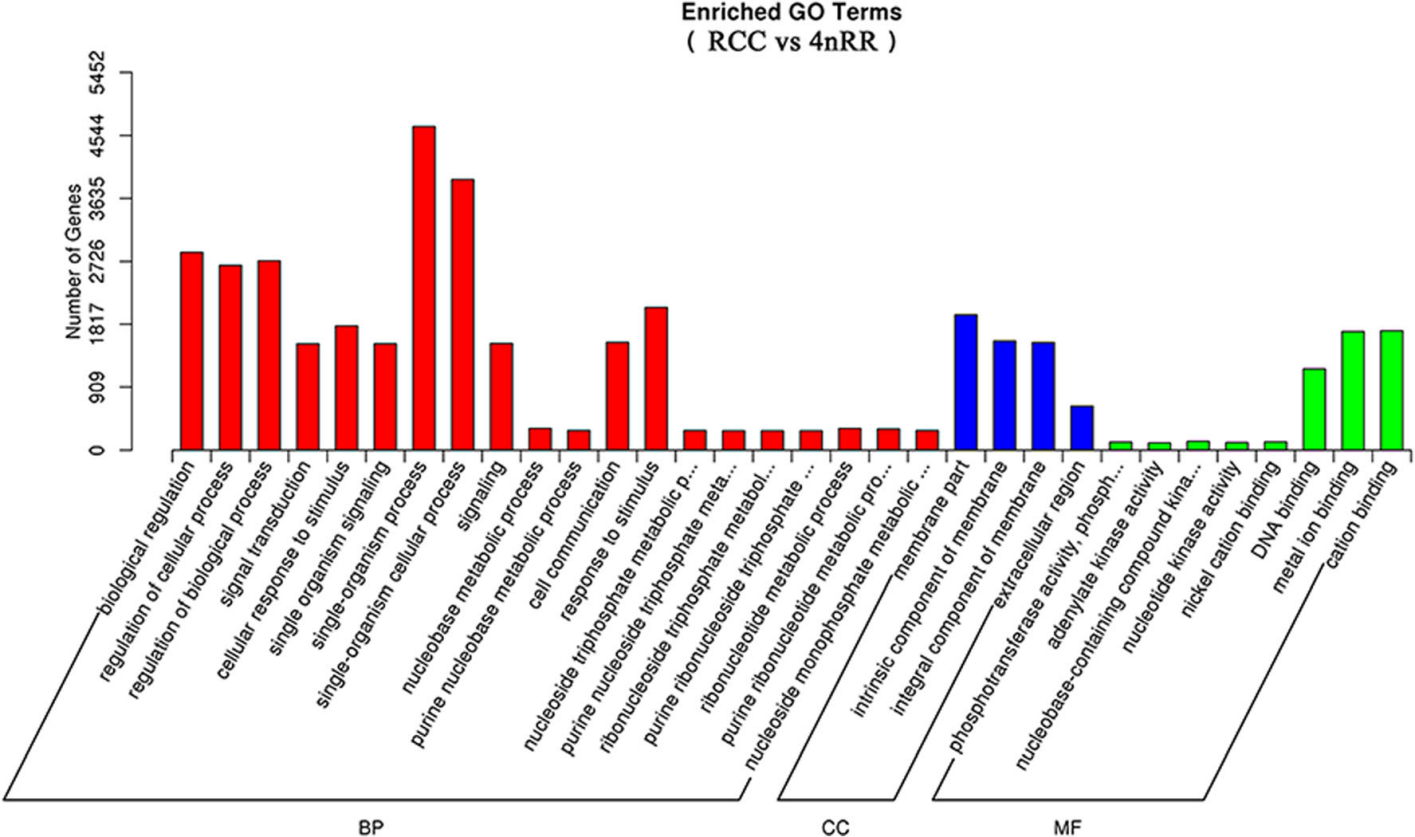
Gene ontology function classification of RCC and 4nRR transcriptome. Gene ontology (GO) term assignments to RCC, 4nRR unigenes are classified into three GO categories (BP, CC, and MF). The left *y*-axis indicates the number of genes. BP, biological processes; CC, cell components; MF, molecular functions. RCC, *Carassius auratus* red var.; 4nRR, autotetraploid hybrid

**Fig. 6:**
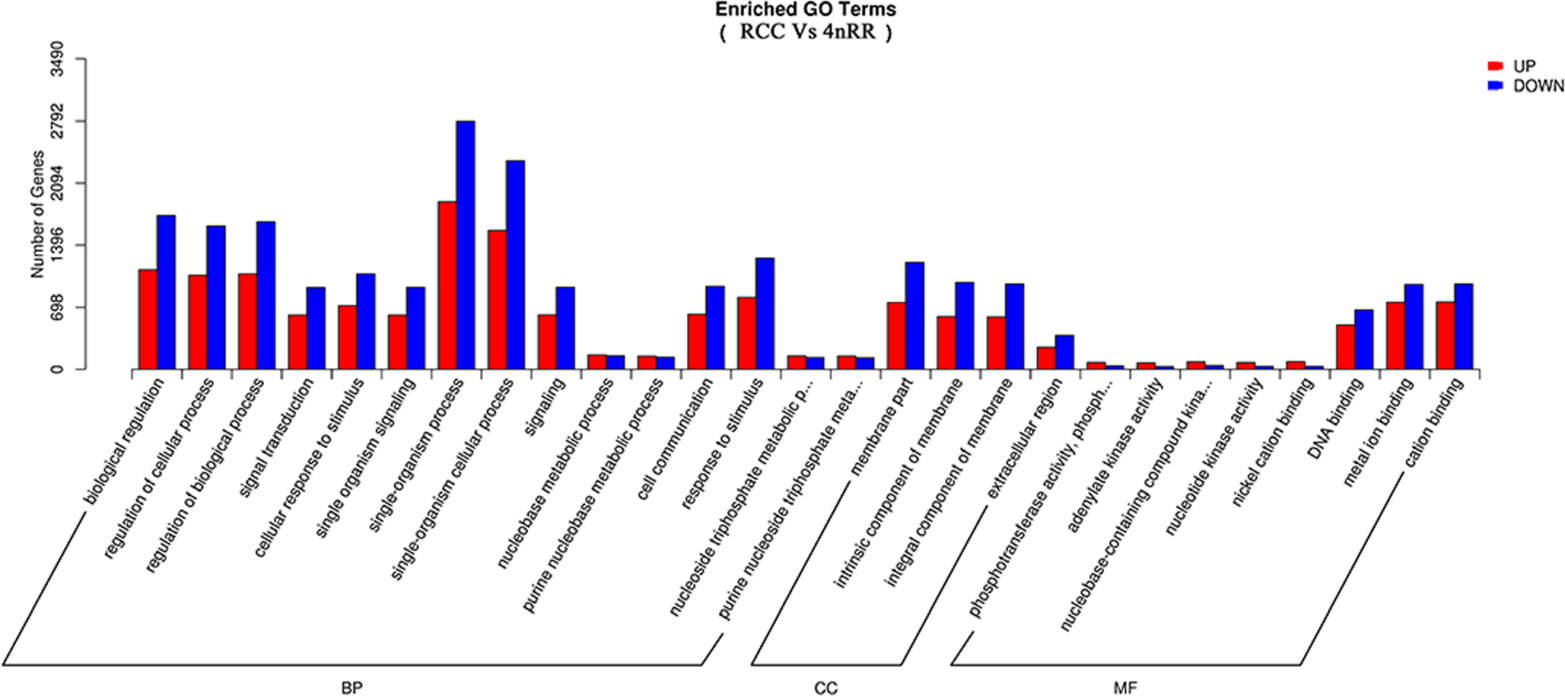
Gene ontology enrichment analysis of RCC and 4nRR transcriptome. Up (red) and down (blue) unigenes were quantified. The comparison results between RCC and 4nRR unigenes are shown in three function/processes. The left *y*-axis indicates the number of genes. BP, biological processes; CC, cell components; MF, molecular functions. RCC, *Carassius auratus* red var.; 4nRR, autotetraploid hybrid

To evaluate the impact of autotetraploidization on gene expression, we compared the ovary tissue transcriptome of 4nRR (*F*_1_) with its diploid progenitor (RCC). Each sample contained three biological replicates. The detected genes, as described in 4nRR (*F*_1_) and RCC information resource representations, covered almost all important functional groups of gene ontology (GO) biological processes, molecular functions, and cell components (Fig. 5). Furthermore, an analysis of significant GO group enrichment refined this overview and identified under- and over-representation related to various functions/processes (Fig. 6). Transcriptome analysis identified 8210 genes (4637 up- and 3573 down-regulated) that showed significant changes in mRNA expression (*q* value < 0.005 and |log2(foldchange)| > 1) between 4nRR (F_1_) and RCC (Dataset S1). GO functional analysis showed that several of the most highly differentially expressed genes were related to biological regulation, regulation of cellular process, cellular response to stimulus, and single-organism processes (Dataset S2).

## Discussion

Because of incompatibility between parental chromosomes, allopolyploidization can increase genomic changes (Pontes et al. 2004; Skalická et al. 2005; Gaeta et al. 2007; Lim et al. 2008; Xiong et al. 2011; Qin et al. 2015b). The frequency of genomic change has been associated with divergence of the diploid parental genomes (Song et al. 1995). 4nRB (RRBB, 4n = 148) was formed by combining the two diploid genomes from RCC (RR, 2n = 100) and BSB (BB, 2n = 48), and a large number of genomic changes occurred in the newly established allotetraploid genome (Qin et al. 2015b; Qin et al. 2016). Theoretically, homologous chromosomes should have high compatibility in autotetraploids, but substantial loss of chromosomal loci (centromeric, *5S rDNA*, and Ag-NORs) was observed in first-generation autotetraploid lineages. Moreover, the largest number of nsSNPs and InDels also suggested significant changes in genomic structure, clearly reflecting instability in the newly established autotetraploid genomes. Our data showed that autotetraploidization can also result in rapid and widespread genomic changes. Importantly, these changes lead to divergence between homologous chromosomes and an increase in heterozygosity, which may contribute to stable inheritance and the successful establishment of autotetraploid lineages.

Multivalent pairing inhibits the formation of diploid gametes during meiosis in polyploids, whereas bivalent pairing is considered advantageous for maintaining genetic stability in polyploids (Paterson et al. 2004; Parisod 2010). Consequently, successful polyploidy must maintain diploid-like behavior (Comai et al. 2000; Wendel 2000; Soltis et al. 2009). Indeed, autopolyploids usually exhibit random pairing of chromosomes (non-preferential pairing), where each chromosome has more than one potential partner, which might result in multivalent formation during meiosis (Soltis and Soltis 2000; Wu et al. 2001). However, in this study, each generation of autotetraploids produced diploid gametes and exhibited chromosome number (or ploidy) stability over consecutive generations. In addition, comparative analysis based on 4nRR (*F*_1_) somatic and gamete chromosomal loci revealed obvious chromosomal locus number halving in gametes. This suggests that the coexistence of four homologous chromosome sets does not result in multivalent formation during meiosis in autotetraploid lineages, and diploid-like chromosome pairing was restored. The molecular basis of diploid-like chromosome pairing remains unclear, but we speculate that rapid and widespread genomic changes in newly established autotetraploid genomes might generate immediate and non-random divergence between homologous chromosomes, providing a physical basis for diploid-like chromosome pairing and driving the diploidization process.

Polyploidization not only had a significant effect on genomic architecture (De Bodt et al. 2005; Soltis and Soltis 2009) but also impacted other genetic features, including gene expression (Osborn et al. 2003; Comai 2005). Studies of differential gene expression and transcriptomics have mainly focused on allotetraploids. Transcriptional profiling demonstrated considerably altered transcriptomes in allopolyploids compared with their diploid progenitors (Kashkush et al. 2002; Adams et al. 2004; Wang et al. 2006; Ni et al. 2009). It is generally expected that the uniform genomes of autopolyploids, in contrast to those of allopolyploids, should not exhibit significant gene expression alterations (Zheng et al. 2010). In this study, however, a comparative analysis of transcriptomes between autotetraploids (*F*_1_) and RCC at the same developmental stage identified 8711 transcribed genes, of which 8210 exhibited significant up- or down-regulation, indicating that significant gene expression alterations are observed in newly synthesized autopolyploids. As allopolyploids arise from interspecies hybrids, considerable transcriptome alterations are likely to be caused by the reunion of previously diverged regulatory hierarchies. For autopolyploids, the mechanisms that cause transcriptome alterations are not well understood. We speculate that substantial genomic changes in newly established autotetraploid genomes generate robust divergence between homologous chromosomes, which damage the uniform genomes of autopolyploids and lead to significant transcriptome changes.

## Electronic supplementary material

ESM 1 (DOCX 14 kb)

ESM 2 (DOCX 23 kb)

ESM 3 (DOCX 14 kb)

ESM 4 (DOCX 14 kb)

ESM 5 (XLSX 532 kb)

ESM 6 (XLSX 230 kb)
