# Correction to: Rapid Genomic and Genetic Changes in the First Generation of Autotetraploid Lineages Derived from Distant Hybridization of *Carassius auratus* Red Var. (♀) × *Megalobrama amblycephala* (♂)

**DOI:** 10.1007/s10126-018-09872-9

**Published:** 2019-01-18

**Authors:** Qinbo Qin, Liu Cao, Yude Wang, Li Ren, Qiwen Liu, Yuwei Zhou, Chongqing Wang, Huan Qin, Chun Zhao, Shaojun Liu

**Affiliations:** State Key Laboratory of Developmental Biology of Freshwater Fish, College of Life Sciences, Hunan Normal University, Changsha, 410081 Hunan People’s Republic of China

**Correction to: Marine Biotechnology**

10.1007/s10126-018-9859-8

The original version of this article was revised twice due to the following changes:

(1) The article “Rapid Genomic and Genetic Changes in the First Generation of Autotetraploid Lineages Derived from Distant Hybridization of *Carassius auratus* Red Var. (♀) × *Megalobrama amblycephala* (♂)”, written by Qinbo Qin, Liu Cao, Yude Wang, Li Ren, Qiwen Liu, Yuwei Zhou, Chongqing Wang, Huan Qin, Chun Zhao, and Shaojun Liu, was originally published electronically on the publisher’s internet portal (currently SpringerLink) on 13 November 2018 with open access.

With the author(s)’ decision to step back from Open Choice, the copyright of the article changed on January 2019 to © Springer Science+Business Media, LLC, part of Springer Nature 2018 and the article is forthwith distributed under the terms of copyright.

(2) The article "Rapid Genomic and Genetic Changes in the First Generation of Autotetraploid Lineages Derived from Distant Hybridization of Carassius auratus Red Var. (♀) × Megalobrama amblycephala (♂)", written by Qinbo Qin, Liu Cao, Yude Wang, Li Ren, Qiwen Liu, Yuwei Zhou, Chongqing Wang, Huan Qin, Chun Zhao, and Shaojun Liu, was originally published electronically on the publisher’s internet portal (currently SpringerLink) on 13 November 2018 without open access.

With the author(s)’ decision to opt for Open Choice the copyright of the article changed on February 2019 to © The Author(s) 2018 and the article is forthwith distributed under the terms of the Creative Commons Attribution 4.0 International License (http://creativecommons.org/licenses/by/4.0/), which permits use, duplication, adaptation, distribution and reproduction in any medium or format, as long as you give appropriate credit to the original author(s) and the source, provide a link to the Creative Commons license and indicate if changes were made.

The original article has been corrected.

