# Supplementary material for: Rapid Genomic and Genetic Changes in the First Generation of Autotetraploid Lineages Derived from Distant Hybridization of Carassius auratus Red Var. (♀) × Megalobrama amblycephala (♂)
Source: Mar Biotechnol (NY). 2018 Nov 13;21(2):139–49. doi: 10.1007/s10126-018-9859-8 (PMC6441405; doi:10.1007/s10126-018-9859-8)
Supplement: Supplementary file 1 — (DOCX 14 kb) [file 10126_2018_9859_MOESM1_ESM.docx]

**Table S1. The basic information of genome sequencing**.

|  | 4nRR(F_1_) |
| --- | --- |
| Raw reads (Million) | 1,015.87 |
| Clean reads (Million) | 819.33 |
| Clean base (Gb) | 103.22 |
| Q20 (%) | 92.86 |
| Q30 (%) | 86.30 |
| GC (%) | 37.70 |

^a^ Coverage ratio represent that the ratio of reference genome region was mapped by the sequencing data.
