# Supplementary material for: Rapid Genomic and Genetic Changes in the First Generation of Autotetraploid Lineages Derived from Distant Hybridization of Carassius auratus Red Var. (♀) × Megalobrama amblycephala (♂)
Source: Mar Biotechnol (NY). 2018 Nov 13;21(2):139–49. doi: 10.1007/s10126-018-9859-8 (PMC6441405; doi:10.1007/s10126-018-9859-8)
Supplement: Supplementary file 2 — (DOCX 23 kb) [file 10126_2018_9859_MOESM2_ESM.docx]

**Table S2. The function of the top 1000 genes regard with the highest number of non-synonymous single nucleotide polymorphisms (nsSNPs)**

| No. Genes | GO Accession | GO Categories (Level 3) |
| --- | --- | --- |
| 88 | GO:0048856 | anatomical structure development |
| 85 | GO:0007275 | multicellular organismal development |
| 71 | GO:0050789 | regulation of biological process |
| 66 | GO:0050794 | regulation of cellular process |
| 64 | GO:0044238 | primary metabolic process |
| 62 | GO:0044237 | cellular metabolic process |
| 62 | GO:0009653 | anatomical structure morphogenesis |
| 57 | GO:0043170 | macromolecule metabolic process |
| 56 | GO:0007154 | cell communication |
| 44 | GO:0048869 | cellular developmental process |
| 34 | GO:0007155 | cell adhesion |
| 31 | GO:0006807 | nitrogen compound metabolic process |
| 26 | GO:0051234 | establishment of localization |
| 25 | GO:0006810 | transport |
| 24 | GO:0009790 | embryonic development |
| 23 | GO:0032989 | cellular component morphogenesis |
| 22 | GO:0065008 | regulation of biological quality |
| 20 | GO:0009058 | biosynthetic process |
| 19 | GO:0006928 | cell motion |
| 19 | GO:0006996 | organelle organization |
| 19 | GO:0051674 | localization of cell |
| 18 | GO:0006950 | response to stress |
| 16 | GO:0019222 | regulation of metabolic process |
| 16 | GO:0065009 | regulation of molecular function |
| 15 | GO:0030030 | cell projection organization |
| 15 | GO:0050793 | regulation of developmental process |
| 13 | GO:0007017 | microtubule-based process |
| 13 | GO:0050878 | regulation of body fluid levels |
| 13 | GO:0048870 | cell motility |
| 12 | GO:0003008 | system process |
| 12 | GO:0035074 | pupation |
| 12 | GO:0009605 | response to external stimulus |
| 12 | GO:0033036 | macromolecule localization |
| 11 | GO:0048646 | anatomical structure formation involved in morphogenesis |
| 11 | GO:0051239 | regulation of multicellular organismal process |
| 10 | GO:0007049 | cell cycle |
| 10 | GO:0051641 | cellular localization |
| 9 | GO:0051716 | cellular response to stimulus |
| 9 | GO:0022607 | cellular component assembly |
| 9 | GO:0048523 | negative regulation of cellular process |
| 9 | GO:0007389 | pattern specification process |
| 9 | GO:0048519 | negative regulation of biological process |
| 9 | GO:0051649 | establishment of localization in cell |
| 8 | GO:0048518 | positive regulation of biological process |
| 8 | GO:0045184 | establishment of protein localization |
| 7 | GO:0002520 | immune system development |
| 7 | GO:0009056 | catabolic process |
| 7 | GO:0055114 | oxidation reduction |
| 7 | GO:0048522 | positive regulation of cellular process |
| 7 | GO:0009628 | response to abiotic stimulus |
| 6 | GO:0022414 | reproductive process |
| 6 | GO:0008219 | cell death |
| 6 | GO:0016192 | vesicle-mediated transport |
| 6 | GO:0048647 | polyphenic determination |
| 6 | GO:0051094 | positive regulation of developmental process |
| 5 | GO:0022402 | cell cycle process |
| 5 | GO:0030029 | actin filament-based process |
| 5 | GO:0043062 | extracellular structure organization |
| 5 | GO:0055085 | transmembrane transport |
| 5 | GO:0051093 | negative regulation of developmental process |
| 5 | GO:0048589 | developmental growth |
| 5 | GO:0048897 | myelination of lateral line nerve axons |
| 5 | GO:0032879 | regulation of localization |
| 5 | GO:0007610 | behavior |
| 5 | GO:0031503 | protein complex localization |
| 4 | GO:0008283 | cell proliferation |
| 4 | GO:0034621 | cellular macromolecular complex subunit organization |
| 4 | GO:0043580 | periplasmic space organization |
| 4 | GO:0043933 | macromolecular complex subunit organization |
| 4 | GO:0003006 | reproductive developmental process |
| 4 | GO:0001503 | ossification |
| 4 | GO:0051128 | regulation of cellular component organization |
| 4 | GO:0051606 | detection of stimulus |
| 3 | GO:0007163 | establishment or maintenance of cell polarity |
| 3 | GO:0016044 | membrane organization |
| 3 | GO:0019725 | cellular homeostasis |
| 3 | GO:0007632 | visual behavior |
| 3 | GO:0035265 | organ growth |
| 3 | GO:0040008 | regulation of growth |
| 3 | GO:0070271 | protein complex biogenesis |
| 2 | GO:0019953 | sexual reproduction |
| 2 | GO:0006955 | immune response |
| 2 | GO:0031294 | lymphocyte costimulation |
| 2 | GO:0044236 | multicellular organismal metabolic process |
| 2 | GO:0022613 | ribonucleoprotein complex biogenesis |
| 2 | GO:0046931 | pore complex biogenesis |
| 2 | GO:0051301 | cell division |
| 2 | GO:0034994 | microtubule organizing center attachment site organization |
| 2 | GO:0009892 | negative regulation of metabolic process |
| 2 | GO:0051129 | negative regulation of cellular component organization |
| 2 | GO:0052192 | movement in environment of other organism during symbiotic interaction |
| 2 | GO:0002682 | regulation of immune system process |
| 2 | GO:0040012 | regulation of locomotion |
| 2 | GO:0044087 | regulation of cellular component biogenesis |
| 2 | GO:0044089 | positive regulation of cellular component biogenesis |
| 2 | GO:0048583 | regulation of response to stimulus |
| 2 | GO:0048584 | positive regulation of response to stimulus |
| 2 | GO:0051130 | positive regulation of cellular component organization |
| 2 | GO:0009607 | response to biotic stimulus |
| 2 | GO:0009719 | response to endogenous stimulus |
| 2 | GO:0042221 | response to chemical stimulus |
| 2 | GO:0051707 | response to other organism |
| 1 | GO:0032504 | multicellular organism reproduction |
| 1 | GO:0001909 | leukocyte mediated cytotoxicity |
| 1 | GO:0031640 | killing of cells of another organism |
| 1 | GO:0002252 | immune effector process |
| 1 | GO:0002253 | activation of immune response |
| 1 | GO:0006066 | alcohol metabolic process |
| 1 | GO:0006323 | DNA packaging |
| 1 | GO:0006413 | translational initiation |
| 1 | GO:0008037 | cell recognition |
| 1 | GO:0032940 | secretion by cell |
| 1 | GO:0034330 | cell junction organization |
| 1 | GO:0051651 | maintenance of location in cell |
| 1 | GO:0070193 | synaptonemal complex organization |
| 1 | GO:0022415 | viral reproductive process |
| 1 | GO:0022411 | cellular component disassembly |
| 1 | GO:0043954 | cellular component maintenance |
| 1 | GO:0007276 | gamete generation |
| 1 | GO:0007618 | mating |
| 1 | GO:0009566 | fertilization |
| 1 | GO:0048609 | reproductive process in a multicellular organism |
| 1 | GO:0043480 | pigment accumulation in tissues |
| 1 | GO:0048771 | tissue remodeling |
| 1 | GO:0048871 | multicellular organismal homeostasis |
| 1 | GO:0050817 | coagulation |
| 1 | GO:0050879 | multicellular organismal movement |
| 1 | GO:0016203 | muscle attachment |
| 1 | GO:0019827 | stem cell maintenance |
| 1 | GO:0031076 | embryonic camera-type eye development |
| 1 | GO:0048066 | pigmentation during development |
| 1 | GO:0002684 | positive regulation of immune system process |
| 1 | GO:0031341 | regulation of cell killing |
| 1 | GO:0031343 | positive regulation of cell killing |
| 1 | GO:0032844 | regulation of homeostatic process |
| 1 | GO:0043900 | regulation of multi-organism process |
| 1 | GO:0043902 | positive regulation of multi-organism process |
| 1 | GO:0045927 | positive regulation of growth |
| 1 | GO:0051240 | positive regulation of multicellular organismal process |
| 1 | GO:0051235 | maintenance of location |
| 1 | GO:0051656 | establishment of organelle localization |
| 1 | GO:0009856 | pollination |
| 1 | GO:0044419 | interspecies interaction between organisms |
