# Supplementary material for: Rapid Genomic and Genetic Changes in the First Generation of Autotetraploid Lineages Derived from Distant Hybridization of Carassius auratus Red Var. (♀) × Megalobrama amblycephala (♂)
Source: Mar Biotechnol (NY). 2018 Nov 13;21(2):139–49. doi: 10.1007/s10126-018-9859-8 (PMC6441405; doi:10.1007/s10126-018-9859-8)
Supplement: Supplementary file 3 — (DOCX 14 kb) [file 10126_2018_9859_MOESM3_ESM.docx]

**Table S3. The distribution of the SV (DEL, INS and INV) in 4nRR (F_1_)**

|  | Categories | Exon | Intron | Intergenic region | Total |
| --- | --- | --- | --- | --- | --- |
| RCC | DEL | 5,133 | 40,008 | 44,434 | 89,575 |
|  | INS | 10,515 | 14,484 | 17,473 | 42,472 |
|  | INV | 1,187 | 1,369 | 2,209 | 4,765 |
