# Supplementary material for: Rapid Genomic and Genetic Changes in the First Generation of Autotetraploid Lineages Derived from Distant Hybridization of Carassius auratus Red Var. (♀) × Megalobrama amblycephala (♂)
Source: Mar Biotechnol (NY). 2018 Nov 13;21(2):139–49. doi: 10.1007/s10126-018-9859-8 (PMC6441405; doi:10.1007/s10126-018-9859-8)
Supplement: Supplementary file 4 — (DOCX 14 kb) [file 10126_2018_9859_MOESM4_ESM.docx]

**Table S4. The statistical data of insertion or the deletion of bases in coding sequences**

|  | RCC |
| --- | --- |
| Insertion in CDS | 24,112 |
| Deletion in CDS | 20,159 |
| Heterozygosity in CDS | 2,342 |
| Homozygosity in CDS | 41,929 |
| Total in CDS | 44,271 |
| Insertion in Genome | 3,025,151 |
| Deletion in Genome | 2,637,200 |
| Heterozygosity in Genome | 1,142,628 |
| Homozygosity in Genome | 4,519,723 |
| Total in Genome | 5,662,351 |
